# Scalable computation of ultrabubbles in pangenomes by orienting bidirected graphs

**DOI:** 10.1093/bioinformatics/btag235

**Published:** 2026-07-07

**Authors:** Juha Harviainen, Francisco Sena, Corentin Moumard, Aleksandr Politov, Sebastian Schmidt, Alexandru I Tomescu

**Affiliations:** Department of Computer Science, University of Helsinki, Helsinki, Finland; Department of Computer Science, University of Helsinki, Helsinki, Finland; Department of Computer Science, University of Helsinki, Helsinki, Finland; Department of Computer Science, ENS de Lyon, Lyon, France; Department of Computer Science, University of Helsinki, Helsinki, Finland; Department of Computer Science, University of Helsinki, Helsinki, Finland; Department of Computer Science, University of Helsinki, Helsinki, Finland

## Abstract

**Motivation:**

Pangenome graphs are increasingly used in bioinformatics, ranging from environmental surveillance and crop improvement to the construction of population-scale human pangenomes. As these graphs grow in size, methods that scale efficiently become essential. A central task in pangenome analysis is the discovery of variation structures. In directed graphs, the most widely studied such structures, *superbubbles*, can be identified in linear time. Their canonical generalization to bidirected graphs, *ultrabubbles*, more accurately models DNA reverse complementarity. However, existing ultrabubble algorithms are quadratic in the worst case.

**Results:**

We show that all ultrabubbles in a bidirected graph containing at least one tip or one cutvertex—a common property of pangenome graphs—can be computed in linear time. Our key contribution is a new linear-time orientation algorithm that transforms such a bidirected graph into a directed graph of the same size, in practice. Orientation conflicts are resolved by introducing auxiliary source or sink vertices. We prove that ultrabubbles in the original bidirected graph correspond to weak superbubbles in the resulting directed graph, enabling the use of existing linear-time algorithms. Our approach achieves speedups of up to 25× over the ultrabubble implementation in vg, and of >200× over BubbleGun, enabling scalable pangenome analyses. For example, on the v2.0 pangenome graph constructed by the Human Pangenome Reference Consortium from 232 individuals, after reading the input, our method completes in under 3 min, while vg requires >1 hour, and four times more RAM.

**Availability and implementation:**

Our method is implemented in the BubbleFinder tool github.com/algbio/BubbleFinder, via the new ultrabubbles subcommand.

## 1 Introduction

### 1.1 Background

Genomic variations induce specific subgraph structures in genome graphs. For instance, in de Bruijn graphs, single nucleotide polymorphisms typically induce *bubbles* (Fasulo *et al.* 2002, Zerbino and Birney 2008) consisting of two paths of the same length sharing the same start and end vertices. In genome assembly, the most prominent generalization of bubbles are *superbubbles* (Onodera *et al.* 2013). These are defined as acyclic subgraphs of a directed graph with a unique entry vertex from which all internal vertices are reachable, and a unique exit vertex reachable from all internal vertices, such that all edges connecting the superbubble to the rest of the graph are incident to the entry or exit vertices. Superbubbles have numerous applications, see e.g. (Kolmogorov *et al.* 2020, Minkin and Medvedev 2020, Rautiainen *et al.* 2023).

The development of the pangenomic field has led to renewed interest in defining and efficiently computing graph structures capturing genomic variation. A particular focus has also been on directly defining these structures in *bidirected* graphs (or in equivalent structures, such as *biedged graphs*), which innately capture the reverse complementarity of the DNA. In a bidirected graph, bidirected edges have a sign (+ or –) at each endpoint, and e.g. bidirected walks must alternate sign when passing through a vertex.

The “canonical” generalization of superbubbles to bidirected graphs are *ultrabubbles*, introduced by Paten *et al.* (2018). They are analogously defined as subgraphs connected to the rest of the graph by two designated endpoints. Within an ultrabubble, all edges incident at each endpoint must have the same sign internal to the bubble, while edges of the opposite sign connect to the rest of the graph. Furthermore, ultrabubbles are internally acyclic (containing no bidirected walks that start and end at the same vertex) and contain no internal tips (vertices whose incident edges all have the same sign).

### 1.2 Motivation

A significant drawback of ultrabubbles compared to superbubbles is that the currently best algorithm computing all of them takes $O({\left(|V|+|E|\right)}^{2})$ time, in the worst case, for a bidirected graph (V,E) (Paten *et al.* 2018). An implementation finding all ultrabubbles exists in the vg toolkit (Garrison *et al.* 2018), based on first finding a set of *snarls* (a superset of ultrabubbles) of size linear in the size of the graph (a *snarl decomposition*), and then checking which of these snarls is an ultrabubble.

Interestingly, we note that (weak) superbubbles can also be applied to bidirected graphs by considering their “doubled” directed graph: every vertex-side v+ and v− is modeled by a separate vertex, and directed edges are added to and from v+ or v− based on the signs of each bidirected edge incident to *v* (see the Supplementary Material, available as supplementary data at *Bioinformatics* online for details). Conceptually, the Python-based BubbleGun tool by Dabbaghie *et al.* (2022) computes, and merges, pairs of matching (weak) superbubbles in this doubled graph. While in our experiments we observed that the number of resulting bubbles matches the number of ultrabubbles, to the best of our knowledge we are unaware of a proof that this would lead to an algorithm for computing *all* ultrabubbles (in fact, such proof would immediately imply that ultrabubbles can be computed in linear time). However, an innate drawback of this approach is that it works on a graph of double the size, leading to double the memory and the time needed to compute superbubbles in it (slowdown which we also confirm experimentally).

Recently, other types of bubble-like structures have been proposed, e.g. *bibubbles*, introduced by Li *et al.* (2024) and *panbubbles*, introduced by Bhat *et al.* (2025), none of which admit linear-time algorithms computing all such structures. These have been shown useful in capturing biological events in complex gene graphs. On the other hand, *snarls*, also introduced by Paten *et al.* (2018), have been recently shown to admit a linear-time algorithm identifying them all, via SQPR trees (Sena *et al.* 2025).

As pangenomes grow, scalability becomes paramount, making linear-time algorithms essential. For example, Release 1 (May 2023) of the Human Pangenome Reference Consortium (HPRC) graph contained 47 individuals (94 haplotypes) and 110 million edges (Liao *et al.* 2023). Release 2 (May 2025) expanded to 232 individuals (https://humanpangenome.org/hprc-data-release-2/) and 206 million edges, while Release 3, scheduled for Summer 2026, is expected to include over 350 individuals (https://humanpangenome.org/release-timeline/).

### 1.3 Results

In this paper, we make progress in the direction of obtaining linear-time algorithms for bubble-like structures, if we assume that the pangenome graph has at least one tip (a vertex whose incident bidirected edges all have the same sign), or at least one cutvertex. In such a graph, we prove that all ultrabubbles can be computed in linear time O(|V|+|E|), significantly improving over the existing $O({\left(|V|+|E|\right)}^{2})$-time algorithm by Paten *et al.* (2018). This improves also over the recent method by Zisis and Trom (2026) of verifying which snarls in a given set are ultrabubbles, running in time O(K|V|+(|V|+|E|)), where *K* is the number of given snarls. While the existence of a tip or a cutvertex is limiting in theory, pangenome graphs are rarely tipless, as also mentioned by Bhat *et al.* (2025), and as we also observed in our experiments, where all graphs had either a tip or a cutvertex.

We obtain this result by showing that computing ultrabubbles can be reduced, in linear time, to computing *weak* superbubbles, a type of bubble which functions as a slight relaxation of superbubbles. Our approach relies on a novel algorithm that transforms a bidirected graph into an equivalent directed graph. Starting from a tip or a cutvertex, the algorithm performs a depth-first search that *orients* the bidirected graph. Orientation is achieved by *flipping* the signs incident to some vertices so that every edge has opposite signs at its endpoints; such edges can then be interpreted as directed edges.

We show that ultrabubbles admit such an orientation, and that once oriented they correspond to weak superbubbles in the resulting directed graph. In cases where orientation conflicts arise—namely, edges whose endpoints have the same sign and for which neither endpoint can be flipped—we resolve these conflicts by replacing the problematic edges with edges incident to newly introduced tips. These new tips correspond to sources or sinks in the directed graph. Although sources and sinks are not inside weak superbubbles by definition, the presence of such a conflict implies that the original structure could not have been an ultrabubble.

We further show that the size of the resulting directed graph remains linear in the size of the original bidirected graph (and having only at most 0.2% additional vertices in the HPRC graphs), that ultrabubbles in the bidirected graph correspond to weak superbubbles in the directed graph, and that essentially all weak superbubbles in the directed graph correspond to ultrabubbles in the bidirected graph. Since ultrabubbles are the canonical generalization of superbubbles (Paten *et al.* 2018), such a relationship must have existed in principle, although it had not previously been made explicit.

Our orientation algorithm is also very simple and efficient. We implemented it into our BubbleFinder tool (Sena *et al.* 2025) (via the new subcommand ultrabubbles), and we used the direct linear-time weak superbubble-finding algorithm by Gärtner and Stadler (2019), via its existing C++ implementation. On the HPRC Release 1 and Release 2 pangenome graphs in GBZ format, after reading the input (with the same parsing library), BubbleFinder is 25× faster than vg, while using four times less RAM (i.e. 14 GiB for Release 1 and 24.8 GiB for Release 2 graphs). On the HPRC Release 1 graph in GFA format, BubbleFinder is 200× faster than BubbleGun, using three times less RAM.

## 2 Materials and methods

### 2.1 Preliminaries

A *bidirected graph* G=(V,E) has vertex set V=V(G) and bidirected edge set E=E(G). A *sign* is a symbol α∈{+,−}, and the *opposite sign* $\hat{\alpha }$ of α is defined as $\hat{+}=-$ and $\hat{-}=+$. A pair (v,α) where v∈V(G) and α∈{+,−} is a *vertex-side* (Rahman and Medvedev 2022) [this is also called *signed vertex* in (Abrishami *et al.* 2025)], which we concisely write as vα, e.g. v+ or v−. A *bidirected edge* e∈E(G) is an unordered pair of vertex-sides {uα,vβ}; for simplicity we may refer to bidirected edges as just edges. We say that *G* has a vertex-side of sign α in *u* or that *u* has sign α over *e*. The set of vertex-sides of *G* is the set $\underset{e\in E(G)}{\cup }e$. We let $N_{G}^{+}(v)$ (resp. $N_{G}^{-}(v)$) denote the set of those vertices *u* for which there is an edge {v+,uα} (resp. {v−,uα}) in *G*. We say that a bidirected graph *H* is a *subgraph* of *G* (and write H⊆G) if V(H)⊆V(G) and E(H)⊆E(G) (we also say that *G* is a *supergraph* of *H*). We say that *v* is a *tip* in *G* if no two vertex-sides of *G* have distinct signs in *v*; a vertex *v* is a *source* (resp. *sink*) if all the vertex-sides of *G* have sign + (resp. –) in v. The subgraph *induced* by a subset C⊆V(G) of vertices of *G* is the graph with vertex set *C* and the subset of edges in E(G) whose endpoints are in *C*, which is denoted by *G[C]*.

**Figure 1:**
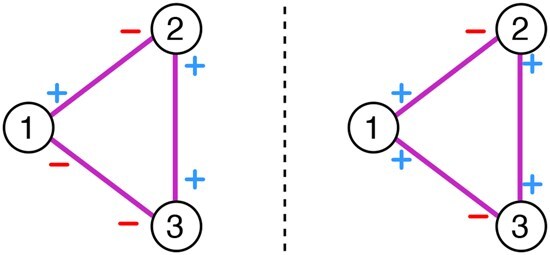
Examples of cycloids in bidirected graphs. On the left, the cycloid {1+,2−},{2+,3+},{3−,1−}, where the sign alternates at every vertex. On the right, the cycloid {1+,2−},{2+,3+},{3−,1+}, where vertex 1 is the only vertex where the sign does not alternate (i.e. the exceptional vertex). In this paper, we say that a bidirected graph is acyclic if it has no cycloid.

A walk *W* in *G* is a sequence of edges $\left\{s_{1}\alpha_{1},s_{2}\alpha_{2}\right\}$, $\left\{s_{2}{\hat{\alpha }}_{2},s_{3}\alpha_{3}\},\ldots ,\{s_{k-1}{\hat{\alpha }}_{k-1},s_{k}\alpha_{k}\}\in E(G\right)$. We say that *W* is an $s_{1}\alpha_{1}$*-*$s_{k}\alpha_{k}$*walk* or just an $s_{1}$*-*$s_{k}$ walk. Vertices $s_{1}$ and $s_{k}$ are the *first* and *last* vertices of the walk, respectively, and all remaining vertices are its *internal* vertices. Notice that to any walk *W* in a bidirected graph we can associate another walk consisting of the sequence of edges of *W* written in reversed order (i.e. walks in bidirected graphs have two possible orientations). A *path* is a walk without repeated vertices. A *cycloid* is a path where the first and last vertex coincide and where at most one vertex (informally, the *exceptional* vertex) has the same sign over the two edges incident to it in the sequence (see Fig. 1). A graph with no cycloid is *acyclic*. A vertex *w* is a *u-v cutvertex* in a bidirected graph *G* if every path between *u* and *v* in the underlying undirected graph of *G*, i.e. if every *undirected path* between *u* and *v*, contains *w* and w≠u,v. Graph *G* is *biconnected* if for any two vertices u,v∈V(G) there is no *u*-*v* cutvertex (such a vertex can also be simply called a *cutvertex* of *G*). Directed graphs can be seen as a special case of bidirected graphs where every edge {uα,vβ} has α≠β. More precisely, a *directed graph D* has vertex set V(D) and *directed edges* in V×V. A directed edge from *u* to *v* is denoted as *uv* or (u,v), and a *directed path* from $u_{1}$ to $u_{k}$ in *D* is a sequence of edges $u_{1}u_{2},u_{2}u_{3},\ldots ,u_{k-1}u_{k}\in E(D)$.

Let v∈V(G) be a vertex. If $v\alpha_{1},\ldots ,v\alpha_{k}$ are all the vertex-sides of *G* containing *v*, then *flipping v* in *G* amounts to replacing each vertex-side $v\alpha_{i}$ with $v{\hat{\alpha }}_{i}$ for each i∈{1,…,k}; importantly, the identity of the edges of *G* does not change during this process. A bidirected graph is called *digraphic* (Kita 2017) if there is a sequence of flips such that each edge of the resulting graph, say $G^{\prime }$, has vertex-sides of opposite signs. If *D* is the directed graph with the same vertex set as *G* (and $G^{\prime }$) and with edge set $\left\{uv:\{u+,v-\}\in E(G^{\prime })\right\}$, then we say that *D* is an *orientation* of *G*.

Without loss of generality we assume that the underlying undirected graph of our bidirected graphs consist of a single component, since in general we can independently apply our techniques to each component of an arbitrary bidirected graph.

Definition 1 [Superbubbloid and Superbubbloid component (Gärtner *et al.* 2018, Gärtner and Stadler 2019)]. *Let G be a directed graph. Let* (u,v)*be an ordered pair of distinct vertices of G. Then* (u,v)*is a superbubbloid with entry u and exit v if:*

- *reachability: G has a directed path from u to v.*
- *matching: the set of vertices reachable from u without using v as an internal vertex coincides with the set of vertices reaching v without using u as an internal vertex. The graph B is the induced subgraph of G by this set of vertices and it is called the superbubbloid component.*
- *acyclicity: B is acyclic.*

**Figure 2:**
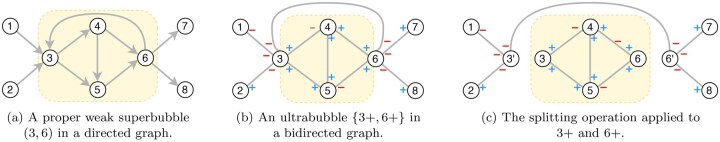
Examples of (weak) superbubbles and ultrabubbles. (a) A proper weak superbubble (3,6) with component having vertex set {3,4,5,6} that is highlighted. Without the edge (6,3), the weak superbubble (3,6) becomes a superbubble. (b) An ultrabubble {3+,6+} with ultrabubble component having vertex set {3,4,5,6} that is highlighted. Note that the signs at 3 and 6 are partitioned according to the ultrabubble component. (c) Applying the splitting operation. For 3+ we add a new node $3^{\prime }$ such that all +-incident edges remain at 3 and all −-incident edges move to $3^{\prime }$ (and analogously for 6+).

We also need the following notions introduced in (Gärtner *et al.* 2018, Gärtner and Stadler 2019). A *weak superbubbloid* is a pair (u,v) with u≠v such that (u,v) satisfies conditions (a) and (b) of superbubbloid [say, with graph *B* from condition (b)], and such that B−vu is acyclic. A *(weak) superbubble* is a (weak) superbubbloid that is minimal in the sense that there is no vertex w∈V(B) distinct from *v* such that (u,w) is a (weak) superbubbloid (see Fig. 2a). A *trivial (weak) superbubble* (u,v) is a (weak) superbubble whose vertex set is {u,v}. For clarity, notice that any superbubbloid is also a weak superbubbloid since *vu* may not be an edge.

To define ultrabubble we need one more definition. The *splitting* operation receives a bidirected graph *G* and a vertex-side uα and produces a new bidirected graph $G^{\prime }=(V^{\prime },E^{\prime })$ with $V^{\prime }:=V(G)\cup \{u^{\prime }\}$ and $E^{\prime }:=E(G)\setminus \{\{u\hat{\alpha },v\beta \}\in E(G)\}\cup \{\{u^{\prime }\hat{\alpha },v\beta \}|\{u\hat{\alpha },v\beta \}\in E(G)\}$. Essentially, every edge of *G* incident to *u* with sign $\hat{\alpha }$ will be incident to $u^{\prime }$ in $G^{\prime }$ instead, and those edges incident to *u* with sign α remain unchanged from *G* to $G^{\prime }$ (see Fig. 2c), so *u* and $u^{\prime }$ become tips of opposite signs in $G^{\prime }$.

Ultrabubbles were defined by Paten *et al.* (2018) in “biedged graphs.” In fact, ultrabubbles are “snarls” (see Paten *et al.* 2018) whose component is acyclic and whose “interior” contains no tips. Originally, Paten *et al.* (2018) defines acyclicity as the absence of closed walks where at most one vertex is allowed to be entered and left with the same sign. Here, for simplicity, we define acyclicity as the absence of cycloids, and is not hard to see that these two notions are equivalent. (A cycloid is a closed walk, and a closed walk that is not a cycloid can be short-circuited to obtain a cycloid.) Further, here we use an equivalent definition of ultrabubble for bidirected graphs based on the definition of “snarl” given in (Sena *et al.* 2025).

Definition 2 [Ultrabubble and Ultrabubble component (Paten *et al.* 2018, Sena *et al.* 2025), see Fig. 2b].

*Let G be a bidirected graph. Let* {uα,vβ}*be a pair of vertex-sides with distinct* u,v∈V(G)*and* α,β∈{+,−}*. Then* {uα,vβ}*is an ultrabubble if:*

- *separable: the graph created by splitting* uα*and* vβ*contains a separate component* X⊆G*containing u and v but not* $u^{\prime }$*and* $v^{\prime }$*. We call X the ultrabubble component of* {uα,vβ}.
- *tipless: no vertex in* V(X)∖{u,v}*is a tip.*
- *acyclic: X is acyclic.*
- *minimal: no vertex-side* wγ*with vertex* w∈X∖{u,v}*is such that* {uα,wγ}*and* $\left\{w\hat{\gamma },v\beta \right\}$*are separable.*

The *interior* of a separable pair of vertex-sides {uα,vβ} with component *K* is the vertex set V(K)∖{u,v}. A *trivial ultrabubble* is an ultrabubble whose component has vertex set {u,v}.

Consider an ultrabubble {uα,vβ} with component *X*. Notice that if $\left\{u\hat{\alpha },v\hat{\beta }\}\in E(G\right)$ then this edge is clearly not contained in *X* due to the splitting operation. Thus, if we are to compute the ultrabubbles of a bidirected graph *G* by the computation of the superbubbles in an orientation of *G*, then we could miss those ultrabubbles with the “back-edge” (see Fig. 2 and the Supplementary Material, available as supplementary data at *Bioinformatics* online for an illustration). This is essentially the reason why we need weak superbubbles, and, indeed, we believe that the appropriate way to see ultrabubbles in directed graphs is through the notion of weak superbubbles (see Theorems 1 and 2).

### 2.2 Algorithm

To ensure the correctness of our algorithm we require the assumption that the input graph has at least one tip. We always make this assumption in what follows, and we present in the Supplementary Material, available as supplementary data at *Bioinformatics* online the case where the graph has no tips, but at least one cutvertex.

**Figure 3:**
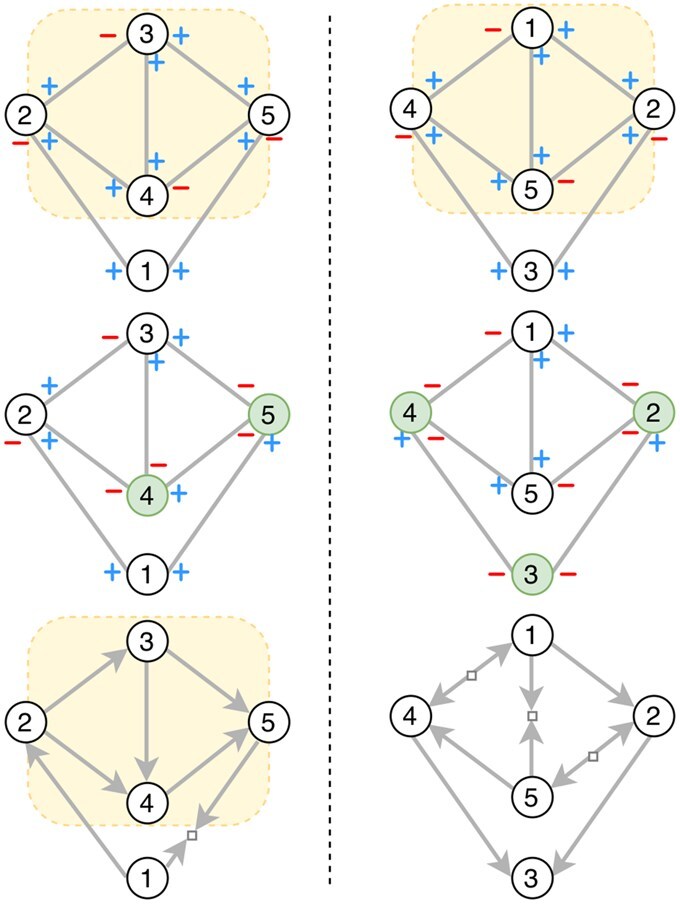
Example of the orientation algorithm on an ultrabubble. The left and right panels show the same graph, but with vertices numbered according to different DFS orders (both starting from vertex 1). On the left, vertex 1 is a tip, while on the right it is a non-tip vertex in the interior of an ultrabubble. Left column. Top: An ultrabubble 2+,5+. Middle: The same ultrabubble after flipping the signs at vertices 4 and 5 (highlighted). The ultrabubble structure is preserved, and all its edges now have opposite signs. Bottom: The directed graph obtained by transforming each edge with opposite signs {u+,v−} into a directed edge from *u* to *v*. Under this transformation, the ultrabubble {2+,5+} becomes the (weak) superbubble (2,5). If the DFS had proceeded from vertex 1 immediately to the vertex now numbered 5, the resulting directed graph would instead contain the (weak) superbubble (5,2), with all edges directed from right to left. Right column. Starting from vertex 1 and following the DFS order that determines the vertex numbering, vertices 2, 3, and 4 (highlighted) must be flipped. However, after these flips it is no longer possible to make all edges of the ultrabubble have opposite signs, which introduces sources and sinks in its interior. Consequently, the ultrabubble {4+,2+} does not correspond to any superbubble in the resulting directed graph.

Algorithm 1 performs a DFS starting from a tip *r* and maintains an array flipped[v]∈{null,true,false} for the vertices v∈V [see Fig. 3 (right) for what can go wrong if we do not start from a tip]. This array serves the purpose of tracking which vertices are flipped and which vertices have already been visited. When arriving at a vertex-side uα, the DFS prioritizes exploring the edges of the form $\left\{u\hat{\alpha },v\beta \right\}$, i.e. it first expands opposite-signed vertex-sides from the one at *u* when it was stacked. Moreover, when reaching an unvisited vertex *v* via an edge {uα,vβ}, *v* is flipped if and only if α=β, so flipped is updated as well as the edges of the graph incident to *v*. As a result, the traversed edge becomes directed (see Fig. 3).

**Figure 4:**
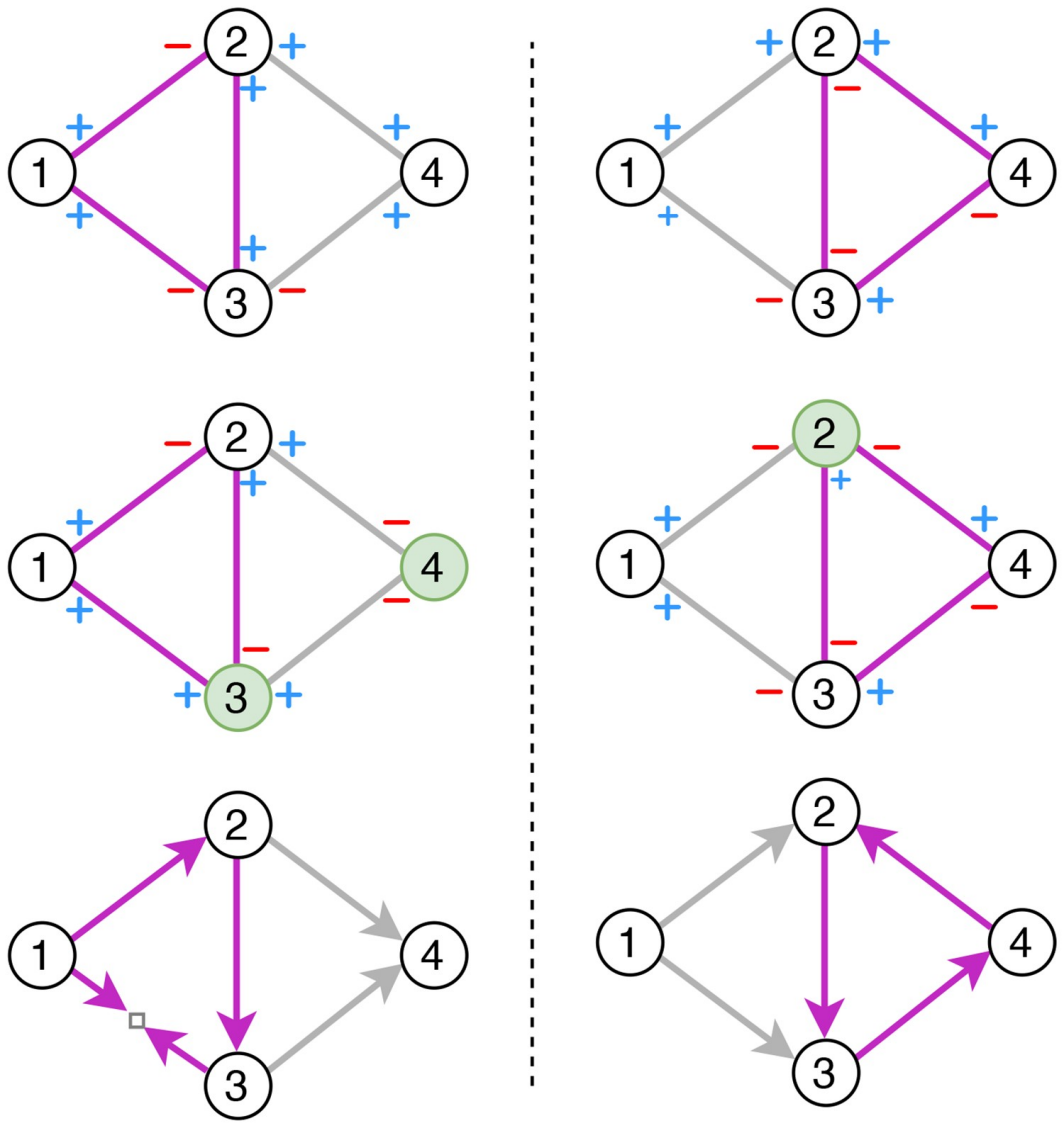
Example of the orientation algorithm on graphs that are not ultrabubbles. On the left column, we have a bidirected graph with a cycloid (highlighted), where only vertex 1 has the same sign at the edges incident to it. On the right column, we have a bidirected graph with a cycloid always alternating signs (highlighted). On the top row, we have the original graphs, with vertices numbered in a DFS order. On the middle row, we have the graph where the vertices in green have flipped signs. For the left-hand side graph, the same-sign edge {2+,3+} requires flipping vertex 3, but then the original edge {1+,3+} cannot be made to have distinct signs because 1 has already been processed and cannot be flipped anymore. As such, we add a fresh vertex in the middle of it, with edges oriented toward the tip. On the right-hand side graph, only vertex 2 needs to be flipped, and no vertices need to be introduced to obtain an equivalent bidirected graph where every edge has opposite signs. The cycle in the original graph maps to the directed cycle in violet.

If later during the search the algorithm scans an equal-signed edge whose endpoints are visited (i.e. a “conflict edge”), this edge is subdivided with a fresh vertex w∈Z such that its two vertex-sides are both “–” if the conflict edge is plus-plus or they are both “+” if the conflict edge is minus-minus (see Fig. 4).

We shall prove that no tip introduced during the DFS is contained in the vertex set of an ultrabubble component and that flipping vertices preserves the set of walks of the graph. These facts together will allow us to show the desired equivalence between ultrabubbles in the original instance and superbubbles in the graph produced by the reduction (with the sole exception that there can be new superbubbles ending or starting at tips introduced by the algorithm, but we do not report these as they do not correspond to ultrabubbles).

In what follows, we denote by *G* the input bidirected graph, by *D* the directed graph produced by Algorithm 1, and by $G^{⋆}$ the bidirected graph that undergoes flips and addition of fresh vertices during Algorithm 1. At the beginning of this algorithm, the vertex set of $G^{⋆}$ (implicitly) coincides with the vertex set of *G*, and the edge set of $G^{⋆}$ (which is denoted by $E^{⋆}$ in Algorithm 1) coincides with the edge set of *G*.

**Algorithm 1** Orienting a bidirected graph **Input** A bidirected graph G=(V,E) containing a tip

**Output** A directed graph D=(V∪Z,A)

 flipped[v]←null, ∀v∈V▹flipped[v]∈{null,true,false}

 Z←∅, $E^{⋆}\leftarrow E$

 **procedure**Flip(*u*) ▹ flip *u* on all edges in $E^{⋆}$

  **for** each edge $e=\{u\alpha ,v\beta \}\in E^{⋆}$**do** 

   $e\leftarrow \{u\hat{\alpha },v\beta \}$

 **procedure**OrientDFS(u,γ) ▹γ is the sign used to enter *u*

  $E_{v}\leftarrow$ the sequence of all edges $\{u\alpha ,v\beta \}\in E^{⋆}$ incident to *u*,

  where those with $\alpha =\hat{\gamma }$ appear before those with α=γ

 **for** each edge $e=\{u\alpha ,v\beta \}\in E_{v}$**do** 

  **if**flipped[v]=null**then** 

  **if**α=β**then** 

   flipped[v]←true

   Flip(*v*)

   $\beta \leftarrow \hat{\beta }$

  **else** 

   flipped[v]←false

  OrientDFS(v,β)

 **else**▹*v* is already fixed, so same-sign edge is a conflict

  **if**α=β**then** 

   Z←Z∪{z}▹*z* is a new vertex

   $E^{⋆}\leftarrow E^{⋆}\setminus e$

   **if**α=+**then** 

    $E^{⋆}\leftarrow E^{⋆}\cup \{\{u+,z-\},\{v+,z-\}\}$

 **else** 

    $E^{⋆}\leftarrow E^{⋆}\cup \{\{u-,z+\},\{v-,z+\}\}$

r← a tip in *G*; flipped[r]←false

OrientDFS(r,+)

A←∅

 **for** each edge $\{x\alpha ,y\beta \}\in E^{⋆}$**do** 

  **if**α=+ and β=−**then**▹α≠β always holds in $E^{⋆}$

   A←A∪{(x,y)}

  **else** 

   A←A∪{(y,x)}

**return** D=(V∪Z,A)

### 2.3 Correctness and complexity

Proofs of the following lemmas can be found in the Supplementary Material, available as supplementary data at *Bioinformatics* online.

Lemma 1. *Let G be a bidirected graph, let* u∈V(G)*be a vertex, and let* $G^{\prime }$*be the graph obtained from G by flipping u. Let W be a sequence of edges in G and let* $W^{\prime }$*be the same sequence of edges in* $G^{\prime }$*. Then W is a walk in G if and only if* $W^{\prime }$*is a walk in* $G^{\prime }$.

The next lemma follows directly by examining Algorithm 1.

Lemma 2. *Algorithm 1 only changes* $G^{⋆}$*by flipping vertices or by adding tips via subdivision of edges.*

Lemma 3. *At the end of Algorithm 1 every edge of* $G^{⋆}$*has opposite signs in its vertex-sides, that is, every edge of* $G^{⋆}$*becomes directed.*

Lemma 4. *Let G be a bidirected graph and let* {uα,vβ}*be pair of vertex-sides with* u≠v*satisfying conditions (a), (b), and (c) of ultrabubbles (Definition 2). Let X denote the component of* {uα,vβ}*. Then* {uα,vβ}*is an ultrabubble if and only if no vertex in the interior of X is a u-v cutvertex in X.*

Corollary 1. *The underlying undirected graph of an ultrabubble component is biconnected.*

In what follows we assume implicitly that Algorithm 1 always produces a directed graph as output (e.g. in the assumptions of some statements). This raises no issues due to Lemma 3.

Lemma 5. *Let G be a bidirected graph and let* {uα,vβ}*be an ultrabubble with ultrabubble component X. Then Algorithm 1 does not subdivide any edge of X.*

Corollary 2. *Ultrabubble components are digraphic.*

Theorem 1 (Ultrabubble ⇒ Weak superbubble).

*Let G be a bidirected graph and let D be the directed graph produced by Algorithm 1. If* {uα,vβ}*is an ultrabubble of G then* (u,v)*or* (v,u)*is a weak superbubble of D.*

**Proof.** 

Let *X* be the component of the ultrabubble {uα,vβ}. Then no edge of *X* is subdivided with a tip by Lemma 5. Thus Lemma 2 implies that the only changes caused by Algorithm 1 to *X* were flipping vertices, and so $X^{⋆}:=G^{⋆}[V(X)]$, B:=D[V(X)], and *X* have the same vertex set. Moreover, Lemma 1 thus implies that *X* and $X^{⋆}$ have the same set of walks. Recall that every edge of $G^{⋆}$ has vertex-sides of opposite signs due to Lemma 3, and so $X^{⋆}$ witnesses that *X* is digraphic.

To show reachability, first notice that a maximal bidirected path *p* in *X* starting at *u* witnesses that *X* has a uα-vβ bidirected path: otherwise, the last vertex x≠v on this path is entered, say, with sign γ∈{+,−}, and since *x* is not a tip any vertex in $N_{G}^{\hat{\gamma }}(x)\neq \emptyset$ is already in *p* due to its maximality, but extending *p* with an edge from *x* to any such a vertex creates a cycloid, a contradiction. Thus $X^{⋆}$ has a uα-vβ path and hence *B* has a directed path from *u* to *v* or from *v* to *u*. Assume without loss of generality in the remainder of the proof that *B* has a directed path from *u* to *v*. Notice that whenever $\left\{u\hat{\alpha },v\hat{\beta }\}\in E(G\right)$ then this edge is not subdivided in $G^{⋆}$ and becomes the edge *vu* in *D*.

For acyclicity we have that *X* is acyclic and thus so is $X^{⋆}$. Since B−vu is an orientation of $X^{⋆}$, B−vu is acyclic. This in turn implies that *u* is the source and *v* is the sink of B−vu.

We now argue on the matching condition. Every vertex y∈V(X) lies in some *u*-*v* path in *B* (consider, e.g. a longest path in B−vu containing *y*; the first and last vertices on this path are *u* and *v*, respectively, since B−vu is a directed acyclic graph with unique source *u* and unique sink *v*). If we show that every out-neighbor of *u* and every in-neighbor of *v* in *D* is a vertex of *B* we are done (i.e. so that the out-reachability of *u* avoiding *v* and the in-reachability of *v* avoiding *u* are confined to *B*). Since *X* is an ultrabubble component, by the separability property we have $N_{G}^{\alpha }(u),N_{G}^{\beta }(v)\subseteq V(X)$. Since flipping vertices only swaps in-neighborhoods with out-neighborhoods, the same neighborhood containment holds in $G^{⋆}$ up to flipping α and β so that $N_{G^{⋆}}^{+}(u),N_{G^{⋆}}^{-}(v)\subseteq V(B)$. Therefore $N_{D}^{+}(u),N_{D}^{-}(v)\subseteq V(B)$.

To see minimality suppose for a contradiction that *B* has a vertex *w* witnessing its non-minimality. If vu∉E(B) then *B* is a superbubbloid that is not a superbubble and so *w* is a *u*-*v* cutvertex in *B* [to see why, see (Sena *et al.* 2025); essentially, an analogous statement to that of Lemma 4 holds in the context of superbubbles]. So *w* is a *u*-*v* cutvertex in $X^{⋆}$ and thus of *X* too, a contradiction to Lemma 4. Otherwise vu∈E(B). Since (u,w) is a weak superbubbloid, say with graph $B^{\prime }$, then $vu\notin E(B^{\prime })$ because $v\notin V(B^{\prime })$. Moreover, $wu\notin E(B^{\prime })$ for otherwise wu∈E(B) and thus *B* has a cycle without *vu*, a contradiction to the definition of weak superbubbloid containing the edge *vu*. So (u,w) is a superbubbloid and by a symmetrical argument we can conclude that (w,t) is a superbubbloid. Thus B−vu is a superbubbloid with *w* witnessing its non-minimality and hence *w* is a *u*-*v* cutvertex in B−vu (see Sena *et al.* 2025). Since $X^{⋆}$ does not contain the edge {v+,u−} due to the splitting operation, *w* is a *u*-*v* cutvertex in $X^{⋆}$ and so *w* is a *u*-*v* cutvertex in *X*, a contradiction to Lemma 4.

Thus, (u,v) is a weak superbubble in *D* if *u* is the source of *B* or (v,u) is a weak superbubble in *D* if *v* is the source of *B*. ▪

Theorem 2 (Weak superbubble ⇒ Ultrabubble).

*Let* G=(V,E)*be a bidirected graph and let D be the directed graph produced by Algorithm 1. Let* (u,v)*be a weak superbubble of D with* u,v∈V*. Let* α,β∈{+,−}*be such that if* flipped[u]=false*then* α=+*, otherwise* α=−*, and if* flipped[v]=false*then* β=−*, otherwise* β=+*. Then* {uα,vβ}*is an ultrabubble of G.*

**Proof.** 

Let *B* be the component of the weak superbubble (u,v). Then V(B)∖{u,v} contains no sources or sinks of *B* by the matching condition and therefore Algorithm 1 did not introduce tips in the edges of G[V(B)] via subdivisions. Thus, by Lemma 2, the DFS only altered G[V(B)] by flipping some of its vertices and so $G^{⋆}[V(B)]$ and G[V(B)] have the same reachability relation by virtue of Lemma 1. Notice also that X:=G[V(B)], $X^{⋆}:=G^{⋆}[V(B)]$ and *B* have the same vertex set.

We argue on the separability. Notice that any directed path in *D* from a vertex not in *B* to a vertex in *B* contains *u* since *u* is the entry of the weak superbubble, and similarly, any path from a vertex in *B* to a vertex not in *B* contains *v* since *v* is the exit of the weak superbubble [see Gärtner *et al.* (2018), Gärtner and Stadler (2019) for a definition of weak superbubble exposing these properties]. Since *B* has a *u*-*v* directed path and trivially $N_{D}^{+}(u),N_{D}^{-}(v)\subseteq V(B)$ and $N_{D}^{-}(u)\setminus \{v\},N_{D}^{+}(v)\setminus \{u\}\subseteq V(D)\setminus V(B)$, it follows that {u+,v−} is separable in $G^{⋆}$ with component $X^{⋆}$. Since $X^{⋆}$ was obtained from *X* only by flipping vertices, it follows that {uα,vβ} is separable in *G*.

We now argue on the acyclicity. Since B−vu is acyclic so is $X^{⋆}$ (notice that $X^{⋆}$ does not contain the potential bidirected edge {v+,u−} due to the splitting operation). Thus *X* is acyclic because *X* does not contain the potential bidirected edge $\left\{v\hat{\beta },u\hat{\alpha }\right\}$.

We are left to show minimality. Notice that X⊆G is the component containing *u* and *v* and not $u^{\prime }$ and $v^{\prime }$ after splitting uα and vβ in *G*, and that $X^{⋆}\subseteq G^{⋆}$ is the component containing *u* and *v* and not $u^{\prime }$ and $v^{\prime }$ after splitting u+ and v− in $G^{⋆}$. Suppose for a contradiction that there is a vertex *w* witnessing the non-minimality of {uα,vβ}, so *w* is a *u*-*v* cutvertex in *X* by Lemma 4. Thus *w* is a *u*-*v* cutvertex in $X^{⋆}$ since subdividing edges and flipping vertices does not create an undirected *u*-*v* path in $X^{⋆}$ avoiding *w*. So if vu∉E(B) then *B* is a superbubble and *w* is a *u*-*v* cutvertex in *B* since *B* has the same set of undirected paths as $X^{⋆}$, a contradiction to the minimality of (u,v) (see Sena *et al.* 2025). Otherwise, if vu∈E(B) then by the same argument used to show minimality in the proof of Theorem 1 we can conclude that *w* is a *u*-*v* cutvertex of B−vu and thus (u,w) is a superbubbloid, a contradiction to the minimality of (u,v).

We can conclude that {uα,vβ} is an ultrabubble of *G*. ▪

Theorem 3. *We can compute all ultrabubbles of a bidirected graph G with at least one tip, or a cutvertex, in time* O(|V(G)|+|E(G)|).

## 3 Implementation and experiments

### 3.1 Implementation

We implemented the new algorithm in our BubbleFinder tool (Sena *et al.* 2025), available at www.github.com/algbio/BubbleFinder, as a new ultrabubbles subcommand. We used the C++ implementation of the weak superbubble algorithm by Gärtner and Stadler (2019) from www.github.com/Fabianexe/clsd. Tips are identified during parsing and serve as starting points for the orientation DFS; for tipless components, cutvertices are identified via Tarjan’s algorithm (Tarjan 1972). The oriented graph is passed to the CLSD weak superbubble algorithm of Gärtner and Stadler (2019). In practice, few conflict vertices are introduced (Supplementary Material, Table 2, available as supplementary data at *Bioinformatics* online): 2691 on HPRC v1.1 (92.9M nodes) and 244 265 on v2.0 (148.3M nodes); the vg graphs, being already directed, produce none.

**Table 2:** Performance comparison on GFA graphs across five tools computing bubble-like structures.^a^

Type	Dataset	vg snarls		BubbleFinder			BubbleFinder (doubled)			BubbleGun		Billi-heuristic	
		Time	Mem	Parsing	Time	Mem	Parsing	Time	Mem	Time	Mem	Time	Mem
MC	HPRC v1.1 Chr. X	36:57	15.5	1:12	**1:17**	11.2	1:08	1:44	11.2	5:27	5.8	3:38	**4.1**
	HPRC v1.1 Chr. Y	1:57	2.1	0:04	**0:06**	**0.67**	0:04	0:14	2.6	1:29	1.9	0:18	1.2
	HPRC v1.1 CHM13 (47 indiv.)	3:14:31	109.9	4:42	**5:53**	55.4	5:07	17:40	99.3	20:05:42	153.5	23:42	**53.1**
	HPRC v2.0 CHM13 (232 indiv.)	TO (24 h)	446.0	48:19	**50:32**	**142.8**	1:46:17	2:10:45	162.2	TO (24 h)	167.3	2:47:10	83.0
pggb	*E. coli* (50 indiv.)	1:44	2.1	0:03	**0:04**	**0.52**	0:02	0:12	2.4	0:49	1.7	0:20	1.1
	Mouse Chr. 19 (17 indiv.)	5:38	7.7	0:09	**0:15**	**2.0**	0:08	0:53	9.6	3:04	7.0	0:52	4.6
	Primate Chr. 6 (14 indiv.)	29:28	38.1	0:47	**1:24**	**11.0**	0:44	5:07	51.5	20:09	39.4	4:18	26.1
	Tomato Chr. 2 (23 indiv.)	2:47	3.1	0:05	**0:07**	**0.88**	0:04	0:19	3.5	1:15	2.7	0:24	1.6
dbg	M. xanthus (10 indiv.)	0:42	1.4	0:02	**0:04**	**0.53**	0:01	0:16	2.5	0:49	1.7	0:31	1.1
vg	1000GP Chr. 1	9:40	18.7	0:26	**1:03**	**6.3**	0:22	4:45	24.3	19:02	19.5	8:17	15.2
	1000GP Chr. 10	5:44	11.4	0:18	**0:40**	**3.9**	0:12	2:42	14.8	10:18	11.9	4:45	9.4
	1000GP Chr. 22	1:31	3.2	0:05	**0:12**	**1.1**	0:03	0:41	4.3	2:35	3.3	1:00	2.6
Pangene	*E. coli* v1.1	< 1 s	0.04	< 1 s	< 1 s	0.06	< 1 s	< 1 s	0.06	< 1 s	0.04	< 1 s	0.01
	M. tb 152 m p0	0:01	0.03	< 1 s	< 1 s	0.06	< 1 s	< 1 s	0.06	< 1 s	0.02	< 1 s	0.00
	M. tb 152 m p1	0:01	0.03	< 1 s	< 1 s	0.06	< 1 s	< 1 s	0.06	< 1 s	0.02	0:01	0.01
	M. tb 152p	0:01	0.03	< 1 s	< 1 s	0.06	< 1 s	< 1 s	0.07	< 1 s	0.02	< 1 s	0.00
	Human 100	0:04	0.07	< 1 s	< 1 s	0.08	< 1 s	< 1 s	0.08	0:04	0.04	N/A	0.01
	Human 100 + 10%	0:04	0.08	< 1 s	< 1 s	0.06	< 1 s	< 1 s	0.08	0:02	0.04	0:01	0.01
	Human 472	0:13	0.15	0:02	0:02	0.06	< 1 s	< 1 s	0.13	0:02	0.04	0:03	0.01
	Human 472 + 10%	0:14	0.15	0:01	0:01	0.06	< 1 s	< 1 s	0.13	0:04	0.04	0:04	0.01

a Times are wall-clock (single-threaded), formatted as h:mm:ss or m:ss. Memory is peak RSS in GiB. For BubbleFinder and BubbleFinder (doubled), the Parsing column reports the parsing part of the runtime (including on-the-fly construction of the internal graph representation) and is included in the Time column. For the doubled mode, this involves building a graph with twice as many vertices. BubbleFinder (doubled) uses the doubled-graph method for ultrabubble detection. Best time and memory per dataset are in **bold** (excluding parsing-only columns and datasets where all tools take under 1 s). TO, exceeded time limit; N/A, tool does not run because the graph is tipless.

BubbleFinder supports GBZ and GFA input formats; for GBZ, it uses the same gbwtgraph library (Sirén and Paten 2022) as vg. BubbleFinder also supports the -T flag of vg to additionally output trivial, i.e. single-edge, ultrabubbles. BubbleFinder also supports finding and merging (weak) superbubbles from the doubled directed graph constructed from the bidirected graph, which is (conceptually) implemented by BubbleGun (see also the Supplementary Material, available as supplementary data at *Bioinformatics* online).

**Figure 5:**
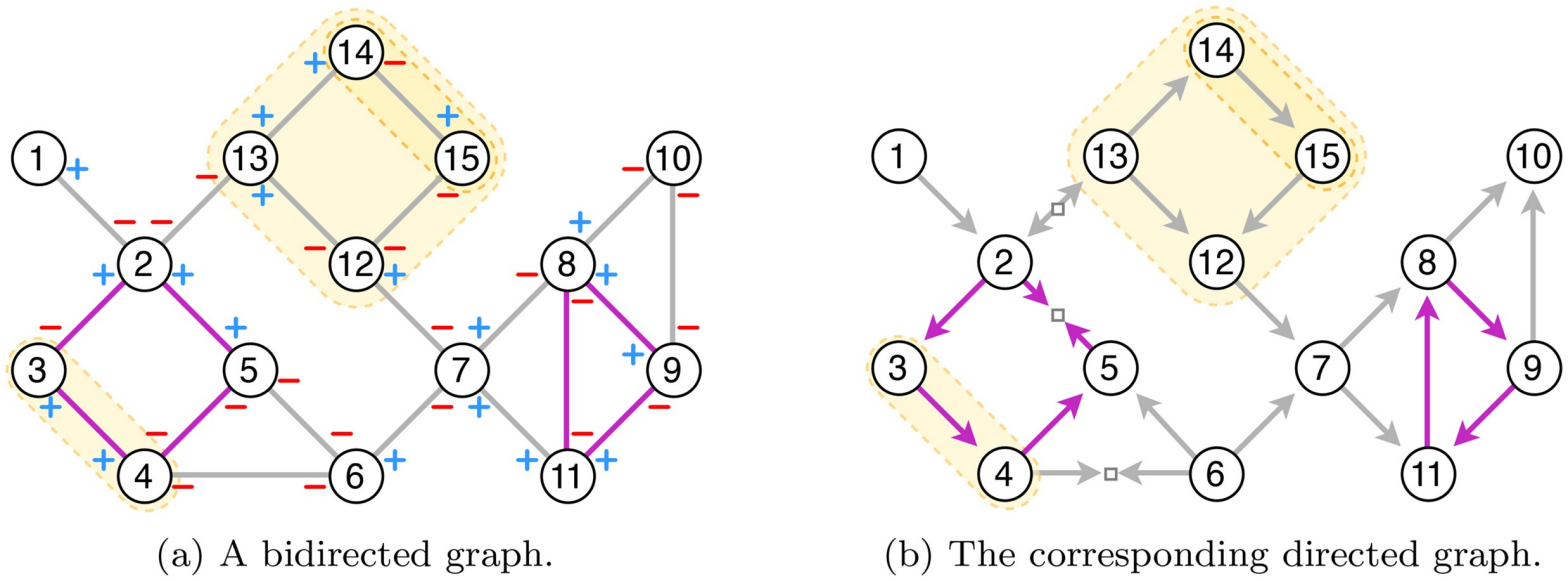
A bidirected graph and the directed graph constructed by Algorithm 1. The graphs contain three ultrabubbles ({3+,4+},{13+,12−},{14−,15+}) and superbubbles ((3,4), (13,12), (14,15)), respectively (no proper weak superbubbles are shown in this figure), highlighted in rounded rectangles. Note that ultrabubble {13+,12−} includes ultrabubble {14−,15+}, and superbubble (13,12) includes superbubble (14,15). The bidirected graph contains a cycloid {2+,3−},{3+,4+},{4−,5−},{5+,2+} with exception at vertex 2, which introduces a sink in the directed graph between vertices 2 and 5, and a cycloid {8+,9+},{9−,11+},{11−,8−} without exceptional vertex, which becomes a directed cycle (highlighted edges). (a) A bidirected graph. (b) The corresponding directed graph.

As an additional feature in BubbleFinder, we also implement the computation of the tree hierarchy of ultrabubbles. This is based on the superbubble decomposition computed by Gärtner and Stadler (2019), which we transform into an ultrabubble decomposition by removing superbubbles whose entrance or exit corresponds to a newly introduced tip and connecting its children to their parent in the tree hierarchy. The resulting *ultrabubble decomposition* forms a rooted forest, see the two nested ultrabubbles in Fig. 5 [akin to the snarl decomposition of Paten *et al.* (2018), only that snarls can also contain cycles or tips].

### 3.2 Experimental results

*Datasets.* We perform experiments on five families of pangenome graphs: (i) MC: Human Pangenome Reference Consortium (HPRC) Minigraph-Cactus (MC) graphs, downloaded from the HPRC data portal (https://data.humanpangenome.org/alignments/), including HPRC v1.1 (47 individuals) and v2.0 (232 individuals); (ii) pggb: *E. coli*, Primate Chr. 6, Tomato Chr. 2 and Mouse Chr. 19 are pangenome graphs constructed with pggb (Garrison *et al.* 2024); (iiii) dbg: a de Bruijn graph constructed from 10 individuals of M. xanthus, used in the evaluation of BubbleGun (Dabbaghie *et al.* 2022); (iv) vg: Chromosome 1|10|22 are variant graphs for human chromosome 1|10|22 constructed from the (∼6.5 millions) variant calls from phase 3 of the 1000 Genomes project 1000 Genomes Project Consortium (2015). The data underlying this article are available in Zenodo, at https://dx.doi.org/10.5281/zenodo.19209715. (v) Pangene: complex gene graphs by Li *et al.* (2024), including *E. coli*, M. tb and Human pangene graphs, used in the evaluation of Billi (Bhat *et al.* 2025). See the Supplementary Material (Table 2, available as supplementary data at *Bioinformatics* online) for statistics on the size of all these graphs, and the numbers of tips and cutvertices.

*Tools.* We compare BubbleFinder with the ultrabubble finding algorithm by Paten *et al.* (2018), available in vg via the subcommand snarls. We also compare with BubbleGun (Dabbaghie *et al.* 2022), and with the panbubble heuristic of Billi (Bhat *et al.* 2025) [we did not use the exact algorithm implemented in Billi, because as observed in Bhat *et al.* (2025), the exact one does not scale to large pangenome graphs, and it has the same behavior as the heuristic version on the smaller graphs tested in Bhat *et al.* (2025)]. We used vg v1.72.0, BubbleGun v1.2.0, and Billi v1.0 (https://github.com/at-cg/billi, commit a77892e). Our snakemake pipeline for the experiments is available at github.com/algbio/BubbleFinder-experiments/. All our experiments ran on Intel(R) Xeon(R) CPU E7-8890 v4 @ 2.20 GHz machines with 1024 GB of RAM.

*Results.* A detailed comparison of ultrabubble counts across all tools is given in the Supplementary Material (Table 1, available as supplementary data at *Bioinformatics* online). On all tested datasets, BubbleFinder reports the same number of ultrabubbles as vg (both trivial and non-trivial). Moreover, also the mode of BubbleFinder of computing and merging superbubbles in the doubled graph reports the same number of bubbles in all the tested graphs. However, this generally uses more than double the time and memory of the orientation-based mode, and to the best of our knowledge there exists no proof that this approach correctly computes the set of ultrabubbles.

**Table 1:** Performance comparison of vg snarls and BubbleFinder, on HPRC graphs in GBZ format.^a^

Type	Dataset	vg snarls		BubbleFinder		Parsing	Speedup
		Time	Mem (GiB)	Time	Mem (GiB)	(GBZ, s)	excl. parsing
MC	HPRC v1.1 Chr. X	2:02	5.1	0:15	1.7	0:10	24.74×
	HPRC v1.1 Chr. Y	0:32	1.7	0:05	0.56	0:04	18.97×
	HPRC v1.1 CHM13 (47 indiv.)	37:23	66.3	3:54	14.3	2:34	25.99×
	HPRC v2.0 CHM13 (232 indiv.)	1:12:13	101.8	9:40	24.8	7:04	25.09×

a Times are wall-clock (single-threaded), formatted as h:mm:ss or m:ss. Memory is peak RSS in GiB. Both tools use the same GBZ parsing library (gbwtgraph). Speedup is computed excluding shared GBZ parsing time.

On the pangenome graphs MC, pggb and vg, the difference between snarls (including trivial) and ultrabubbles (including trivial) is very small. Likewise, the difference between the number of panbubbles computed by Billi-heuristic and non-trivial ultrabubbles is also very small. While the non-ultrabubble subgraphs may capture relevant structures, on these graphs ultrabubbles appear to cover a very large proportion of snarls and panbubbles. On the dbg graph, there are only three more panbubbles than non-trivial ultrabubbles and on the vg graphs all numbers match. On the Pangene graphs the bubble structures are more different, e.g. there are even more panbubbles than snarls on some datasets, and as (Bhat *et al.* 2025) already showed, panbubbles do capture relevant biological events in such cases. However, these graphs are much smaller than pangenome graphs (having at most 42 000 edges). Moreover, notice this dataset contains one tipless graph where Billi cannot run (since it assumes graphs with at least one tip), while this can still be handled by BubbleFinder because it has a cutvertex.

In Table 1, we show the comparison between vg and BubbleFinder on the HPRC graphs in GBZ compressed format (Sirén and Paten 2022) with the -T flag. This format is designed to be parsed much more efficiently than GFA, as we also observe in the “Parsing” column of Table 1 (see also Table 2 for GFA runtimes). Excluding the shared GBZ parsing time, BubbleFinder achieves speedups of 19–26× over vg on the HPRC graphs. On HPRC v2.0, after parsing, BubbleFinder completes in under 3 min while vg requires >1 hour and four times more RAM (101.8 GiB versus 24.8 GiB). In fact, also on the smaller datasets, the memory consumption of BubbleFinder is on par with or lower than that of vg. The 25× speedup combined with the 4-fold reduction in memory footprint highlights the practical advantages of our linear-time orientation approach for population-scale pangenomics. Moreover, we tried to convert the other datasets in GBZ format, but the haplotypes in the GFA file did not cover all edges present in it, and thus we could not obtain equivalent GBZ graphs, and hence omitted the results.

Since BubbleGun and Billi do not support GBZ input, in Table 2 we compare all tools on graphs in GFA format. For completeness, we also compare with vg on these graphs with the -T flag, same as for BubbleFinder. On the v1.1 HPRC graph, our method is 200× faster than BubbleGun, and on the HPRC v2.0, BubbleFinder completed in under 51 min, while BubbleGun timed out. Moreover, BubbleGun seems to report a few cases of bubbles that are not ultrabubbles, see the Supplementary Material, available as supplementary data at *Bioinformatics* online. BubbleFinder is faster also than Billi-heuristic (4× on HPRC v1.1 and 3× on HPRC v2.0), but note that Billi computes more complex structures.

## 4 Conclusion

While bidirected graphs are more expressive than directed graphs—since they naturally capture the reverse complementarity of DNA—we showed that, for the purpose of computing ultrabubbles, they can be oriented to yield an ordinary directed graph (without the need to double the graph, and with a very small number of additional vertices, in practice). Under this orientation, the more general problem of finding ultrabubbles reduces to computing weak superbubbles. This leads to substantial speedups compared to computing (ultra)bubbles with other tools.

Our orientation algorithm raises the broader question of which other variation structures on bidirected graphs can be reduced to directed graphs. Natural candidates include bibubbles (Li *et al.* 2024) and panbubbles (Bhat *et al.* 2025), for which no linear-time algorithms are currently known. Any such orientation reductions would likely need to be substantially more sophisticated than the one presented here, reflecting the greater structural complexity of these objects (e.g. they can contain cycles, but require a stronger reachability property on the bubble vertices). Moreover, unlike for ultrabubbles, there are no known directed counterparts for bibubbles or panbubbles.

## Supplementary Material

btag235_Supplementary_Data

## Data Availability

The data underlying this article are available in Zenodo, at https://dx.doi.org/10.5281/zenodo.19209715.
